# Deficiency in Aryl Hydrocarbon Receptor (AHR) Expression throughout Aging Alters Gene Expression Profiles in Murine Long-Term Hematopoietic Stem Cells

**DOI:** 10.1371/journal.pone.0133791

**Published:** 2015-07-24

**Authors:** John A. Bennett, Kameshwar P. Singh, Zeenath Unnisa, Stephen L. Welle, Thomas A. Gasiewicz

**Affiliations:** 1 Department of Environmental Medicine, University of Rochester Medical Center, Rochester, New York, United States of America; 2 Department of Medicine, University of Rochester Medical Center, Rochester, New York, United States of America; Emory University, UNITED STATES

## Abstract

Dysregulation of hematopoietic stem cell (HSC) signaling can contribute to the development of diseases of the blood system. Lack of aryl hydrocarbon receptor (AhR) has been associated with alterations in gene expression related to HSC function and the subsequent development of a myeloproliferative disorder in aging female mice. We sorted the most primitive population of HSCs with the highest stem cell potential (Long-term, or LT-HSCs) from 18-month-old AhR-null-allele (AhR-KO) and WT mice and analyzed gene expression using microarray to determine alterations in gene expression and cell signaling networks in HSCs that could potentially contribute to the aging phenotype of AhR-KO mice. Comparisons with previous array data from 8-week old mice indicated that aging alone is sufficient to alter gene expression. In addition, a significant number of gene expression differences were observed in aged LT-HSCs that are dependent on both aging and lack of AhR. Pathway analysis of these genes revealed networks related to hematopoietic stem cell activity or function. qPCR was used to confirm the differential expression of a subset of these genes, focusing on genes that may represent novel AhR targets due to the presence of a putative AhR binding site in their upstream regulatory region. We verified differential expression of PDGF-D, Smo, Wdfy1, Zbtb37 and Zfp382. Pathway analysis of this subset of genes revealed overlap between cellular functions of the novel AhR targets and AhR itself. Lentiviral-mediated knockdown of AhR in lineage-negative hematopoietic cells was sufficient to induce changes in all five of the candidate AhR targets identified. Taken together, these data suggest a role for AhR in HSC functional regulation, and identify novel HSC AhR target genes that may contribute to the phenotypes observed in AhR-KO mice.

## Introduction

Hematopoietic stem cells (HSCs) are the source for continuous lifelong replacement of differentiated cells in peripheral blood and the immune system. Loss of mature hematopoietic cells due to normal attrition, infection, radiation/chemotherapy, stress or environmental exposures can stimulate HSCs to divide and differentiate in order to sustain peripheral blood and immune cell populations. However, like all adult stem cells, HSCs are incapable of sustaining an indefinite number of divisions, and prolonged or excessive proliferation can lead to premature exhaustion. There are significant age-related changes in HSCs, and HSC senescence and/or exhaustion may contribute to disease processes that occur at greater frequency with age [1–3]. However, the functional consequences of these changes may only become apparent in response to hematopoietic stressors. Furthermore, age-related altered immune function limits the success of therapies used in the treatment of disorders such as cancer, and further limits life expectancy as well as quality of life in our aging population.

The aryl hydrocarbon receptor (AhR) is a ligand activated transcription factor that was originally identified as a mediator of the toxicity associated with a variety of persistent environmental pollutants such as dioxins (e.g. TCDD) and certain polychlorinated biphenyls (PCBs) [4]. However, cumulative evidence now indicates a significant physiological role of AhR in the immune system, hematopoietic disease, and regulation of HSC function [5–10]. Human exposure to the xenobiotic AhR agonist TCDD and PCBs is associated with increased incidence of lymphoma and leukemia [11, 12], and low dose TCDD promotes lymphoma development in mice [13]. In mouse HSCs, TCDD alters the expression of genes that regulate circadian rythym and genes associated with cell trafficking [9, 14]. The *ex vivo* exposure to AhR antagonists promotes the expansion of human HSCs/progenitors [15]. Similarly, HSCs from AhR null-allele (AhR-KO) mice have abnormally high rates of cell division [5]. Exposure to TCDD alters numbers and functional capacity of murine HSCs [16]. Aging AhR-KO mice develop a myelodysplasia and HSCs exhibit premature exhaustion and decreased self-renewal capacity [6]. Together these studies suggest that AhR may have a role in the regulation of the HSC quiescence-proliferation balance. Consistent with this hypothesis, the *Ahr* gene is down regulated during proliferation and self-renewal of HSCs, and is expressed during quiescence [17].

A recent analysis of mouse-HSC-specific gene expression and the proximal promoters of 322 HSC-enriched genes identified the AhR response element (AHRE) as one of four motifs for the binding of transcription factors (EGR1, SOX4, AhR and STAT1) that control genes critical for HSC function [18]. However, the exact AhR-regulated pathways that control HSC proliferation-balance and development of premature HSC exhaustion and myelodysplasia in AHR-KO mice remain unclear. The goal of the current study was to gain insight into cellular and molecular mechanisms of AhR in HSC aging, and, in particular, AhR-regulated pathways contributing to the altered self-renewal and myelodysplasia observed in aging AhR-KO mice. Understanding these processes will assist in defining mechanisms by which aging, inherited/acquired gene mutations, and/or exposure to environmental contaminants may promote decreased immune function and development of hematopoietic disease. Here, we report the HSC gene expression profiles of 18-month-old AhR-KO and Wild-type mice, and discuss the underlying gene expression changes that may contribute to the HSC functional and disease phenotype observed in KO animals.

## Materials and Methods

### Ethics Statement

All mice were treated in accordance with approved protocols for both handling and experimental procedures at the University of Rochester Medical Center which are set by the University Committee on Animal Resources (UCAR). All lentiviral experiments were carried out in accordance with policies established and approved by the Institutional Biosafety Committee at the University of Rochester. All experiments described in this work were specifically approved by UCAR under protocol number 93-188R. All efforts were taken to minimize animal suffering.

### Mice

Female AhR-KO mice (B6.129-*Ahr* ^*tm1Bra*^ */J)*, [19], were obtained from C. Bradfield (University of Wisconsin) and maintained as a breeding colony in the animal care facility at the University of Rochester. C57BL/6J Wild-type (WT) mice were obtained from Jackson Laboratories and used as experimental controls. All animals used in these studies were 18 months of age, unless specified otherwise.

### Hematological profile of aging mice

Peripheral blood was collected from the retroorbital plexus vein using capillary tube in BD Microtainer tubes with EDTA (Becton, Dickinson and Company). Hematological profiles of these mice were analyzed using a HESKA Hematology Analyzer (HESKA Corporation, Colorado).

### Sorting and microarray analysis of Lin-CD48-CD150+ (SLAM+) cells

Femur and tibia BM cells from female mice were harvested as previously described [5]. Cells for microarray analysis were obtained by fluorescence-activated cell sorting of lineage depleted bone marrow cells, prepared as previously described [6] following staining with fluorochrome-conjugated antibodies against Sca-1 (V450 Clone D7; BD Pharmingen), cKit (PeCy7 Clone 2B8; BD Pharmingen), CD34 (AF700 Clone Ram34; eBiosciences), CD48 (FITC Clone Hm48-1; BD Pharmingen), and CD150 (APC Clone 459911; R&D Systems). Cells were sorted into RLT+ buffer and placed at -80°C for submission to the Functional Genomics Center at the University of Rochester. Total RNA was isolated from sorted SLAM+ LT-HSCs using an RNeasy Mini Kit (Qiagen). RNA was pre-amplified and cDNA was produced using a WT-Ovation PicoSL kit (Nugen). Microarray analysis was performed using Genechip Mouse Gene 2.0 ST Array (Affymetrix) by the Functional Genomics Center. The Iterplier algorithm was used to generate background-subtracted, quantile-normalized signals from the microarray data. Transcripts with a mean difference in expression of at least 1.5 fold (except where stated otherwise) and P<0.05 were examined for network/pathway associations by Ingenuity Pathway Analysis. Top physiological functions were ascribed to the altered set of genes. The microarray data can be accessed through the Gene Expression Omnibus accession number GSE67378. Microarray data were also analyzed using Gene Set Enrichment Analysis (GSEA) as previously described [6].

### qPCR Array validation and pathway analysis

Quantitative mRNA expression was performed using a custom 96-well PrimePCR Array (Bio-Rad, Hercules, California) containing primer assays for a subset of genes found to be altered in the microarray experiments and containing putative AhR transcription factor binding sites. LT-HSCs (SLAM+) were sorted as described above and total RNA was extracted using an RNeasy Mini Kit (Qiagen). cDNA was prepared as described above and 10 ng of cDNA was used in qPCR reactions via a Bio-Rad CFX96 Real Time PCR instrument. Expression of mRNA for each gene was normalized using the expression of HPRT and GAPDH. Values from AhR-KO mice and WT mice were compared according to the 2^-ΔΔCT^ method. CFX Manager Software (Biorad, California) was used for analysis of qPCR data. Differences were considered significant when relative mRNA expression was 1.5 fold higher or lower than WT control with a p-value of less than 0.05. Genes found to be altered according to these criteria were analyzed using Ingenuity Pathway Analysis to determine altered physiological networks and functions, as well as the relationship of those functions and genes to canonical AhR signaling and reported functions from the literature.

### Lentiviral knockdown of AhR

Lineage negative cells were prepared as described above and placed into Stemspan media (Stemcell Technologies, Vancouver BC, Canada) containing 10ng/mL mSCF. Cells were cultured for 24 hrs. The media was aspirated and replaced with DMEM containing lentiviral particles encoding AhR shRNA (TRC0000218025) from The RNAi Consortium at the Broad Institute (Harvard and Massachusetts Institute of Technology). Cells were then allowed to incubate for 24 hours. Fresh viral media was added, and cells were cultured for an additional 24 hours. Successful transduction was confirmed by visualizing GFP expression on a Zoe Fluorescent Cell Imager (Bio-Rad, California). RNA and cDNA preparation were performed as described above, and samples were analyzed by qPCR at the University of Rochester Medical Center Functional Genomics Core facility.

### Western blotting

Hepa1c1c7 cells were cultured in DMEM and transduced as described previously, using virus encoding multiple shRNA sequences. At the end of transduction, culture media was removed, cells washed twice with PBS, and lysed (Reporter Lysis Buffer, Promega, Madison, WI). Lysates were stored at −80°C. Lysate proteins were separated by SDS-polyacrylamide gel electrophoresis (PAGE) (8.4% acrylamide resolving gel) and transferred to polyvinylidene fluoride (PVDF) membrane which was then blocked using 5% nonfat milk in wash buffer (50mM Tris base, 150mM NaCl, and 0.2% Tween 20, pH 7.5). Antibodies used were AhR (mouse monoclonal Rpt-1) and β-actin (rabbit polyclonal, Sigma, St Louis, MO). The appropriate horseradish peroxidase-conjugated secondary antibodies were purchased from Jackson Immunoresearch (West Grove, PA). Proteins were visualized by chemiluminescence using LumiGlo reagents (KPL, Gaithersburg, MD).

### Statistical analyses

Unless otherwise indicated, results were analyzed and plotted using Graphpad Prism (Graphpad Inc, La Jolla, California). When appropriate, a two-tailed Student’s t-test was used to analyze statistical significance. A p-value of less than 0.05 was considered to be statistically significant. For validation using qPCR Arrays, a p-value of less than or equal to 0.01 was considered to be statistically significant. A right-tailed Fisher exact test was used to calculate a p-value to determine the probability that each biological function or disease assigned to the data set was by chance alone.

## Results

### Aged AhR-KO mice have altered cellular composition of peripheral blood

**Fig 1:**
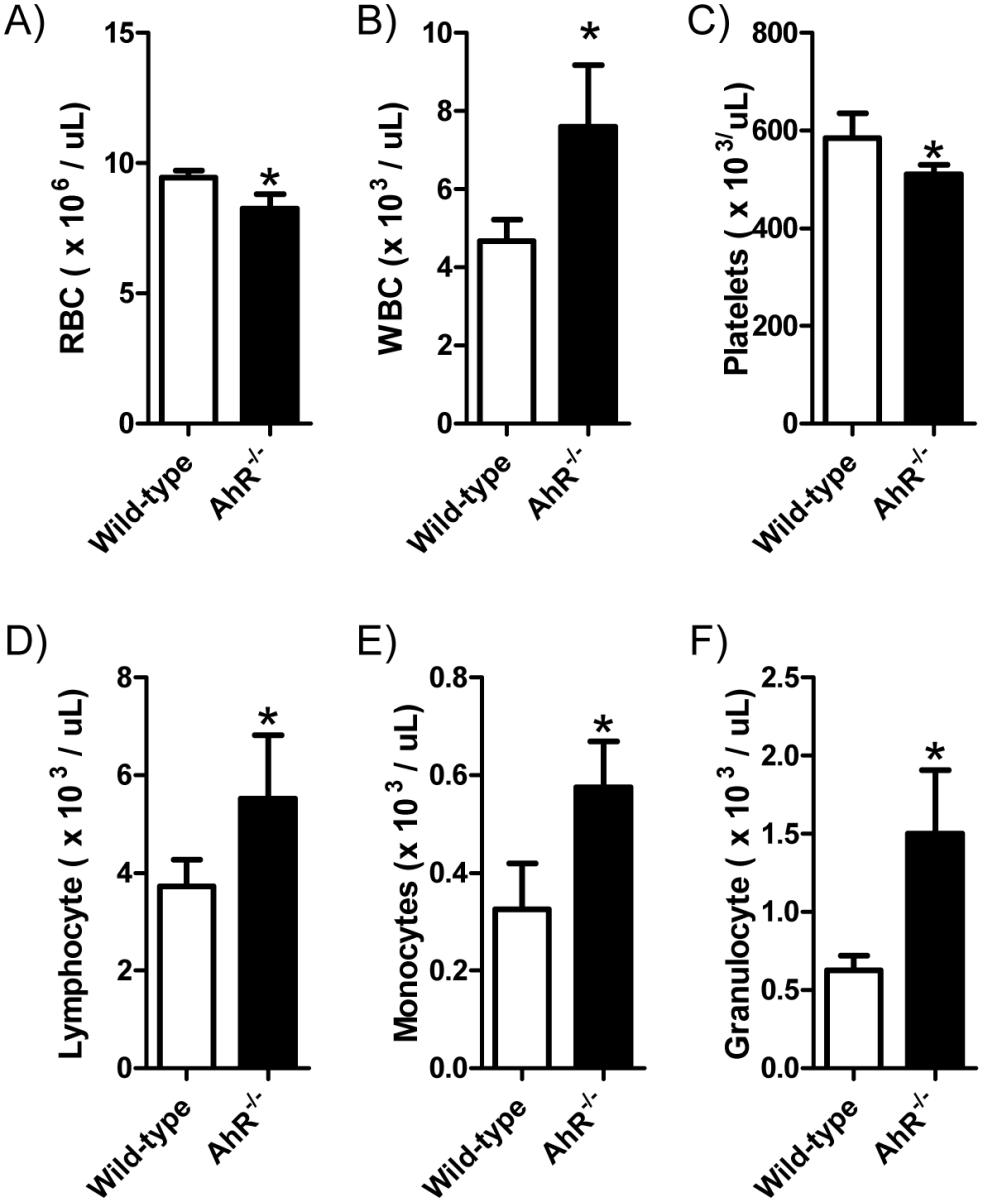
Aged AhR-KO mice have altered composition of peripheral blood. Blood was drawn from the retro-orbital plexus of 18-month-old WT and AhR-KO mice and analyzed for cell counts (A-F). Data are shown as mean +/- S.D. n = 9 mice per group. * values significantly different from WT control (p<0.05)

Our lab previously reported that young AhR-KO mice display a hematopoietic phenotype that includes elevated numbers of white blood cells, alterations in splenic cell populations and increases in the number of phenotypically defined HSC and hematopoietic progenitor populations [5]. Strikingly, even though AhR-KO mice display elevated numbers of LSK cells in bone marrow (a population enriched for stem and progenitor cell potential), these cells fail to engraft irradiated recipient animals as effectively as WT controls in serial transplant assays [5]. These data suggest a defect in AhR-KO LT-HSC function. Aging (18-month-old) AHR-KO mice were also found to have alterations in the composition of peripheral blood; white blood cell counts were nearly two-fold higher in AhR-KO mice and lymphocytes, monocytes and granulocytes were all significantly elevated (Fig 1). Overall, these data indicate that lack of AhR signaling can drive expansion in the hematopoietic compartment and contribute to long-term alterations in hematopoiesis that manifest as changes in peripheral blood composition throughout adult life and into advanced age.

### LT-HSCs from aged AhR-KO mice have significant alterations in gene expression signaling pathways

In order to determine underlying alterations in gene expression and signaling mechanisms in LT-HSCs that could be contributing to the myeloproliferation and decrease in HSC engraftment capability reported in AhR-KO mice, we performed genomic analysis on a population of cells greatly enriched for LT-HSCs. We chose to use 18-month-old mice, as this age represents a time point when maximum levels of myeloproliferation have yet to be achieved [6]; and a time point to determine alterations in gene expression that may be driving further expansion at more advanced ages. LSK CD34- CD48- CD150+ cells from female 18-month-old AhR-KO and WT mice were identified, sorted (S1 Fig) and prepared and submitted for microarray analysis as described above. A list of the top 25 differentially expressed genes in AhR-KO compared to WT are shown in S1 and S2 Tables.

**Fig 2:**
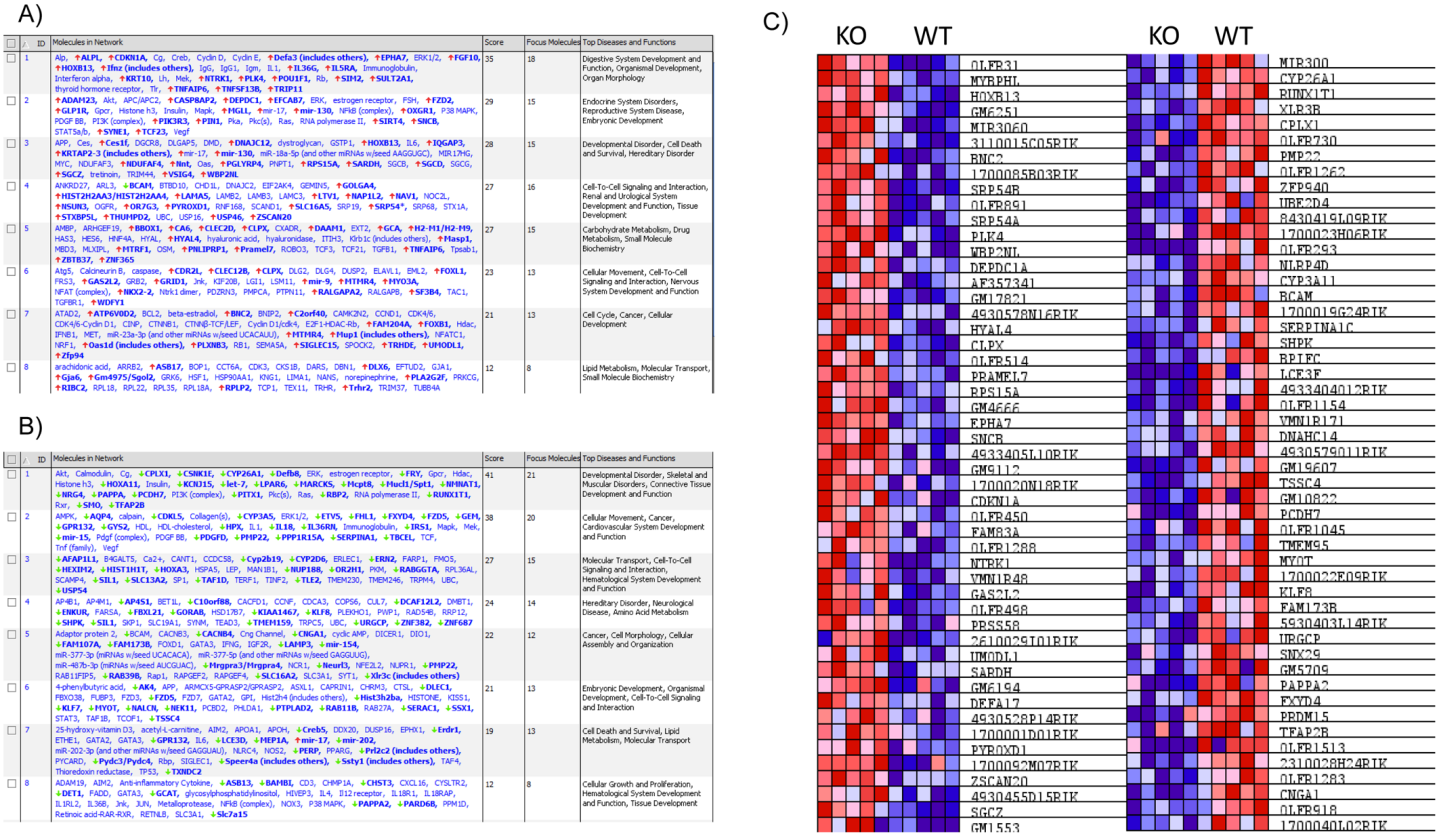
Microarray data was analyzed using Ingenuity Pathway Analysis to determine biological networks that were changed due to lack of AhR. A) Top networks affected with genes found to be up-regulated in LT-HSCs of aged AhR-KO mice relative to aged WT controls. B) Top networks affected with genes found to be down-regulated in LT-HSCs of aged AhR-KO mice relative to aged WT controls. C) Heatmap of top 50 up/down-regulated genes in LT-HSCs of aged AhR-KO mice.

In order to ascribe possible biological functions to the network of gene expression changes observed in LT-HSC (Comparing HSCs from old AhR-KO mice vs. HSCs from old WT mice), data from our microarray were uploaded to Ingenuity Pathway Analysis (IPA). The top biological networks and functions altered in IPA (Fig 2A and 2B) in HSCs lacking AhR include Cell Cycle, Cell Death and Survival, Small Molecule Biochemistry and Drug Metabolism, and Hematological System Development and Function. In addition, a heat map of the top 100 differentially expressed genes was generated (Fig 2C). These networks and changes in gene expression are consistent with known and hypothesized endogenous functions of the AhR in a variety of cell types including HSCs [5, 20–22]. IPA was also used to generate a list of differentially expressed genes (With at least a 1.2 fold change in expression, p<0.05) believed to be related to hematological system development and function. This list included genes such as Akr1c3, FoxL1 and Map3k8 (Table 1).

**Table 1:** Selected genes related to Hematological System Development and Function found to be altered in LT-HSCs of aged AhR-KO mice. This list was generated using Ingenuity software to determine genes related to hematopoietic system function that were altered due to lack of AhR.

Gene name	Expression value	p-value
Il36g	1.848	3.88 x 10^−2^
Il5ra	1.723	2.08 x 10^−2^
Daam1	1.557	2.40 x 10^−2^
Siglec15	1.522	2.21 x 10^−2^
Foxl1	1.516	4.98 x 10^−2^
Trip11	1.512	2.48 x 10^−2^
Tab3	1.498	2.72 x 10^−2^
Tgm1	1.458	2.49 x 10^−2^
Rcan2	1.443	1.42 x 10^−2^
Twf1	1.25	4.79 x 10^−2^
Lgals8	1.229	4.49 x 10^−2^
Prkch	1.218	4.65 x 10^−2^
Slc12a7	-1.307	4.39 x 10^−2^
Sh3bp2	-1.335	2.56 x 10^−2^
Map3k8	-1.345	4.27 x 10^−2^
Slc29a2	-1.398	2.93 x 10^−2^
Aldh1a3	-1.412	4.17 x 10^−2^
Akr1c3	-1.435	4.04 x 10^−2^
Rabggta	-1.586	4.53 x 10^−2^

### Lack of AhR expression and aging cooperatively affect LT-HSC gene expression

**Fig 3:**
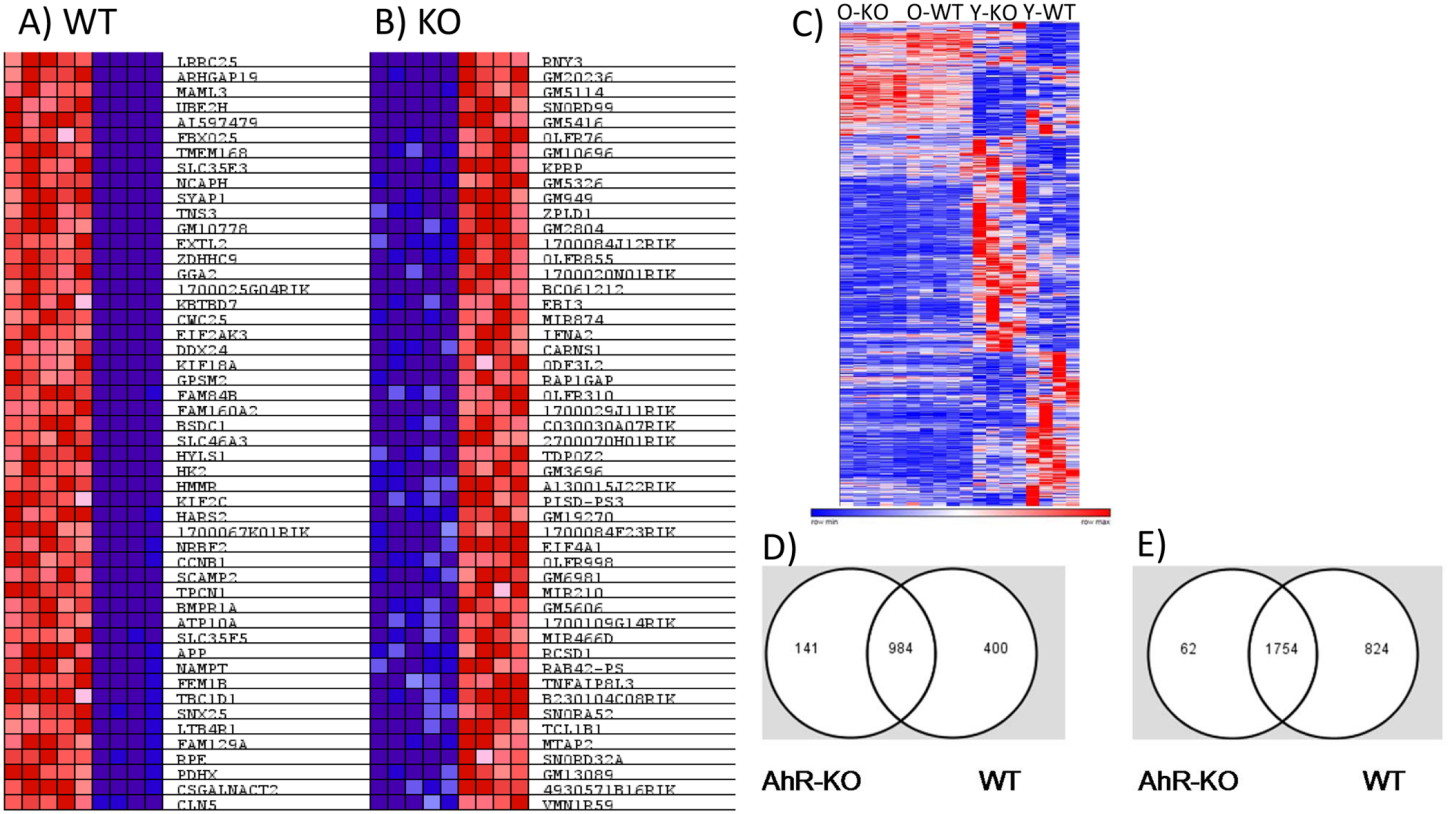
Aging alone changes gene expression in LT-HSCs. Previously published array data on young animals (6) was compared with current data to generate a heat map of top 100 differentially expressed/enriched genes between Old/Young WT (A) and Old/Young AhR-KO (B). (C) Heatmap showing array data for all ages examined. Subsequent analyses focused on genes that require both age and lack of AhR to display differential expression. 203 genes were found to be dependent on lack of AhR and aging, and included 141 up-regulated and 62 down-regulated genes. Overlap of gene changes that occurred in Old/Young WT and Old/Young AhR-KO (D = up-regulated genes, E = down-regulated genes).

**Fig 4:**
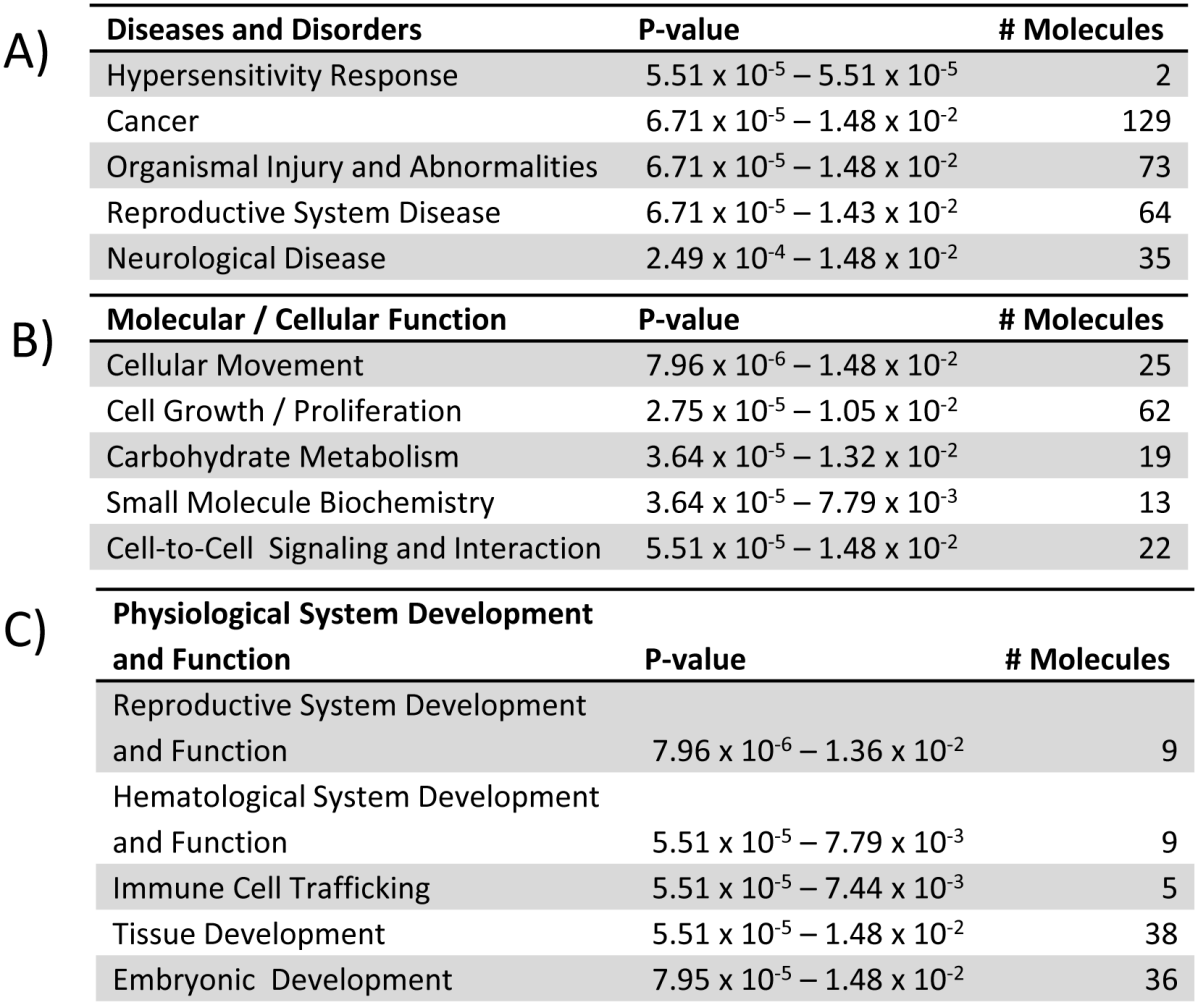
Top Networks and Functions proposed for genes whose differential expression is dependent on both aging and lack of AhR expression. 203 genes which displayed differential expression dependent on both age and lack of AhR were analyzed using IPA to determine top Diseases and Disorders (A), Molecular and Cellular Functions (B), and roles in Physiological System Development and Function (C).

Aging is a significant stressor of the hematopoietic system, and is sufficient to induce changes in gene expression independent of loss of AhR. We sought to determine gene changes related to alterations in HSC function and the myelodysplasia elicited by AhR loss in aging mice [6]. We also identified possible candidates for direct regulation by AhR. To do this, we compared our current array data with our previously published array data from young AhR-KO and WT mice [6] and identified genes that are changed by age alone in WT and AhR-KO animals (Fig 3A and 3B). This list included genes (Bcl2, Ptk2, Nfkb2 and Rb1) previously reported to have altered expression and methylation patterns in aged HSCs [23]. These genes were excluded from further analysis, allowing us to focus on differentially expressed genes dependent on both lack of AhR and increased age. This 203 gene set included 141 genes up-regulated in aged AhR-KO mice, and 62 genes down-regulated in aged AhR-KO mice (Fig 3C, 3D and 3E) that were not altered in aged WT mice. These genes were analyzed by IPA. This analysis indicated that these genes are related to cellular networks and functions such as Cancer, Hematological System Development and Function, and Small Molecule Biochemistry (Fig 4). Genes found to be altered in this analysis included Mrfap1, Gas2l2, Bsph2 and Tfap2b (Table 2). In order to identify candidates that may be directly regulated by AhR, only genes that included a putative AhR binding site in their upstream regulatory region were further analyzed via qPCR. These genes do not appear in Table 2 because their differential expression values were not the highest we observed.

**Table 2:** Top ten up-regulated and top ten down-regulated genes whose differential expression is dependent on lack of AhR expression and chronological age. Ingenuity Pathway Analysis was used to generate a list of genes that display differential expression dependent on both chronological age and lack of AhR expression.

Gene name	Expression value	Age effect p-value
Mrfap1	1.59	4.408 x 10^−2^
Gas2l2	1.56	1.549 x 10^−5^
Slc25a46	1.4	2.565 x 10^−4^
Smc3	1.36	1.696 x 10^−5^
Serpine3	1.34	2.249 x 10^−2^
Rfwd2	1.26	5.812 x 10^−4^
Shisa4	1.25	7.178 x 10^−3^
Gm17769	1.23	8.884 x 10^−3^
Gm19833	1.23	1.928 x 10^−6^
Ppp1cb	1.23	5.000 x 10^−7^
4930524c18Rik	-1.35	1.234 x 10^−4^
Pdzd3	-1.35	3.893 x 10^−5^
Svop	-1.37	8.339 x 10^−5^
Gm12770	-1.43	1.741 x 10^−8^
Inca1	-1.43	2.539 x 10^−3^
Olfr1349	-1.43	5.411 x 10^−6^
2410012e07Rik	-1.47	2.554 x 10^−6^
Irs1	-1.61	1.615 x 10^−5^
Bsph2	-1.89	3.07 x 10^−4^
Tfap2b	-2.5	7.003 x 10^−4^

### qPCR validation of selected genes from microarray

**Fig 5:**
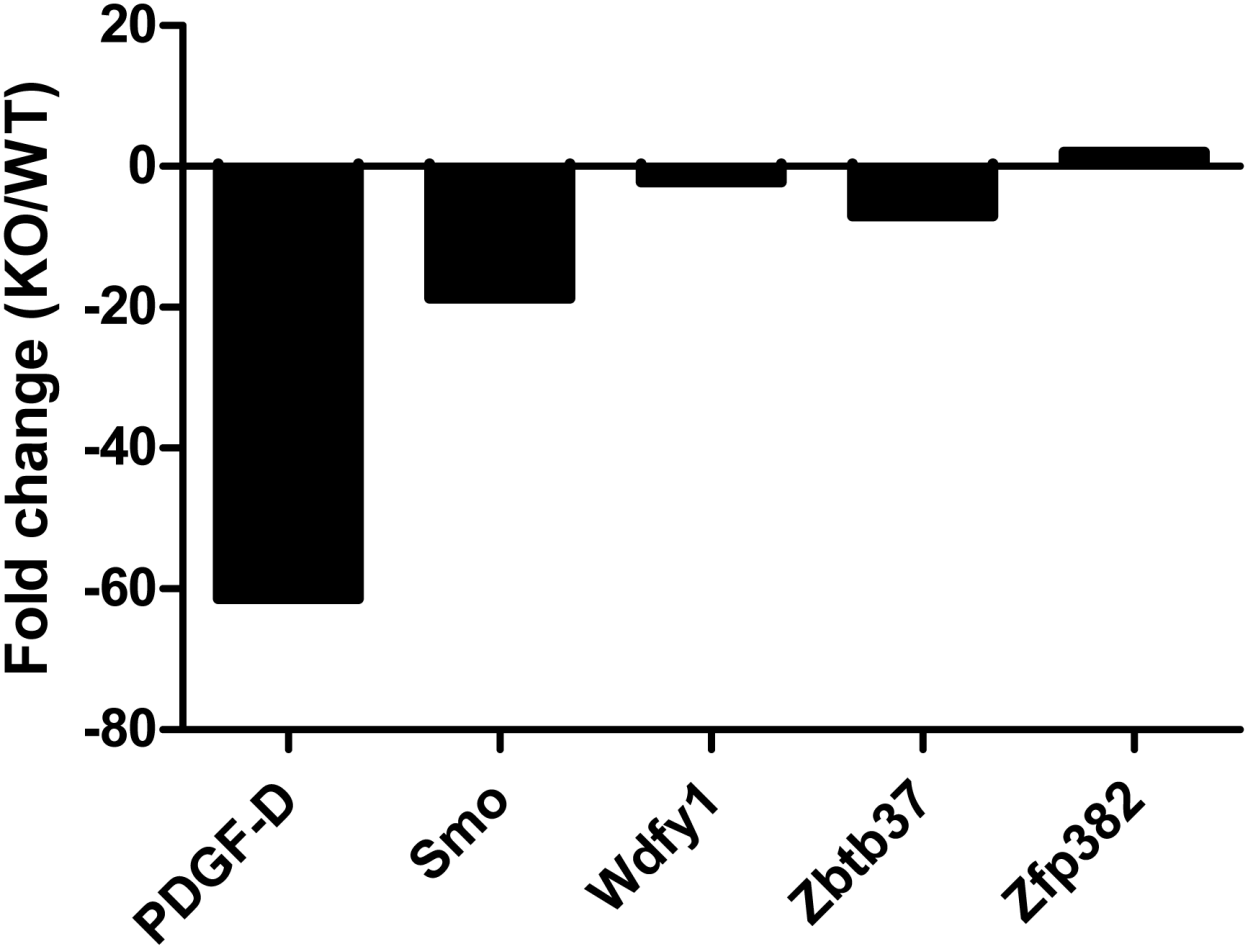
LT-HSCs from AhR-KO mice display alterations in gene expression: LT-HSCs from aged AhR-KO and WT mice were sorted and analyzed for changes in gene expression using qPCR. Genes analyzed were chosen based on their differential expression being dependent on aging as well as lack of AhR expression. The most significant alterations observed were in expression of PDGF-D, Smo, Wdfy1, Zbtb37 and Zfp382. N = 4 each group. A fold change of at least 1.5 was considered significant. Data are fold change values in AhR-KO calculated relative to WT using GAPDH for normalization.

There were 41 genes that were differentially expressed due to age and absence of AhR, which also have putative AHREs in the proximal promoters (determined by Genomatix MatInspector software). A subset of these genes were determined to be differentially expressed by qPCR (Fig 5). The most significant changes observed were in the expression of PDGF-D and Smo. PDGF-D is a member of the platelet-derived growth factor family [24, 25], and its role in hematopoiesis is unknown. Smo has a proposed role in regulating hematopoiesis by interacting with downstream targets Gli1 [26], Gli2 and Gli3 [27]. In addition, Wdfy1, Zbtb37 and Zfp382 were found to be significantly altered. These data identify novel candidates that AhR may directly regulate during chronological aging or proliferative stress of the hematopoietic system/HSCs.

### Novel AhR target genes have predicted functional overlap with AhR

**Fig 6:**
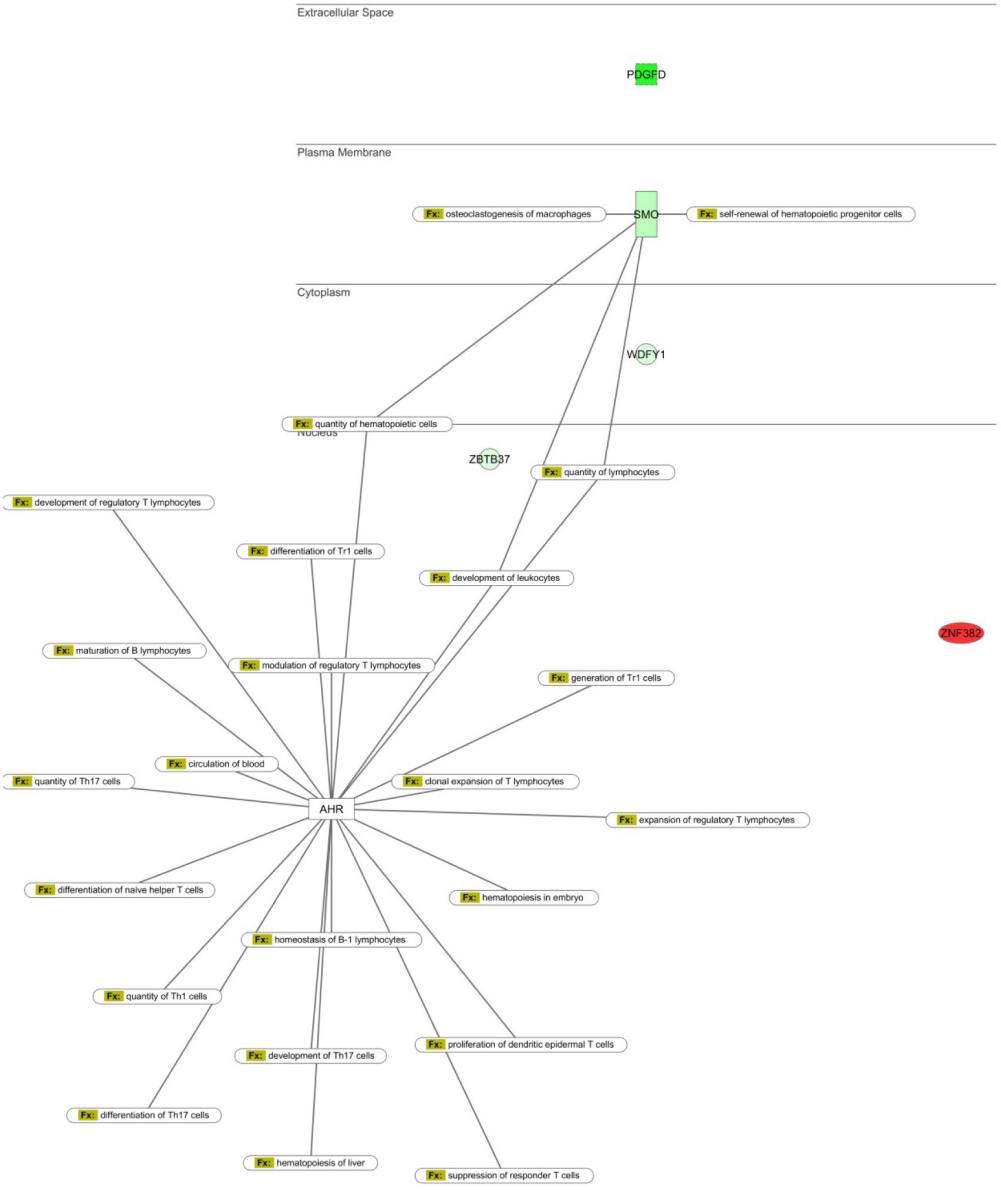
qPCR validated gene changes dependent on lack of AhR and aging in LT-HSCs of AhR-KO mice are related to cell-proliferation, trafficking and HSC function and have functional overlap with AhR. Ingenuity Pathway Analysis was used to determine biological functions of genes verified with qPCR in aged AhR-KO mice (relative to WT controls), and to determine functional overlap with AhR in regulating hematopoiesis.

We again used IPA to determine the possible biological functions and signaling pathway interactions of the subset of genes containing AhR binding sites whose differential expression was verified by qPCR (and their relationship to proposed functions of the AhR). We overlaid pathways generated for Cancer, Cell Proliferation, Hematopoiesis and Cell Migration, and AhR (Fig 6, S2, S3, and S4 Figs). The networks reveal that AhR, PDGF-D, Smo, Wdfy1, Zbtb37 and Zfp382 converge on regulating some of the same functions, including ones directly related to HSC output, such as quantity and self-renewal of hematopoietic cells (Fig 6). Overall these data support the novel hypothesis that AhR may regulate HSC function in part by regulating the expression of PDGF-D, Smo, Wdfy1, Zbtb37 or Zfp382.

### Lentiviral knockdown of AhR is sufficient to change expression of novel AhR target genes

**Fig 7:**
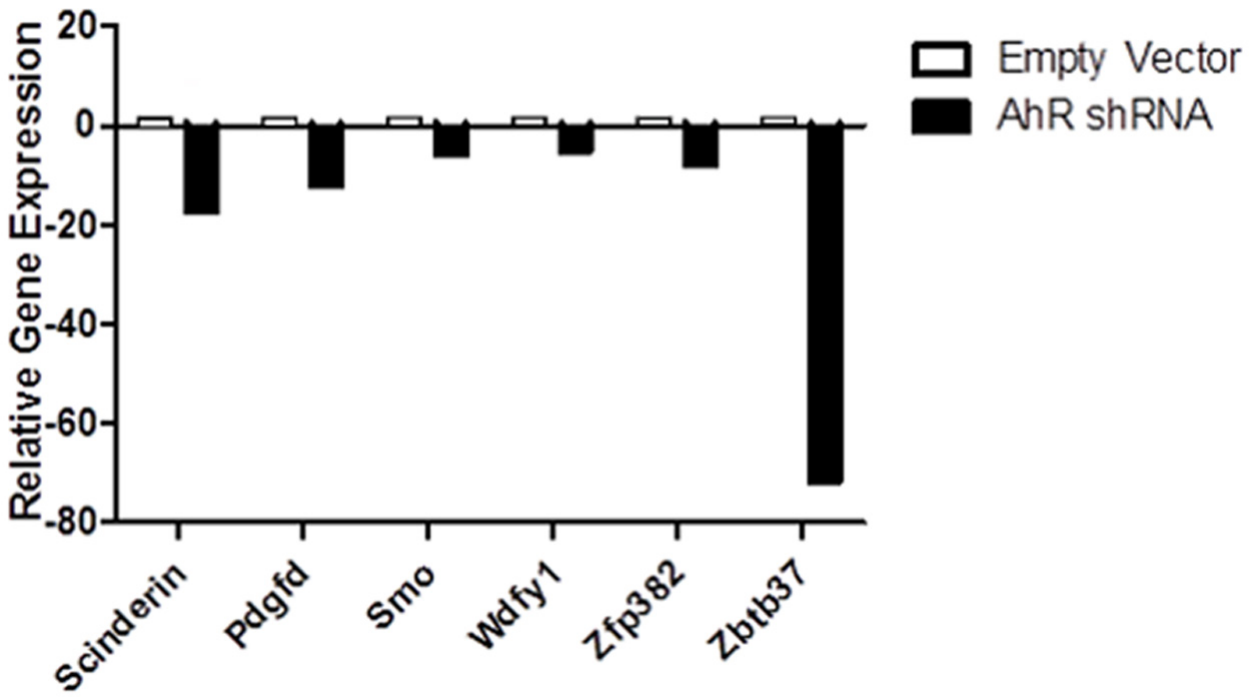
Lentiviral-mediated knockdown of AhR promotes changes in expression in candidate AhR target genes in Lin- hematopoietic cells. Lentiviral vectors encoding TRC0000226025 (AhR-shRNA) were used to transduce primary murine lin- cells. A fold change of at least 1.5 was considered significant. Relative gene expression was calculated using GAPDH and HPRT as endogenous controls. Data expressed as samples transduced with viral particles encoding AhR-shRNA relative to empty vector control. A fold change of at least 1.5 was considered significant. N = 4 per group.

In order to test the hypothesis that AhR is a direct regulator of PDGF-D, Smo, Wdfy1, Zbtb37 or Zfp382, we utilized a lentiviral vector to deliver AhR shRNA to primary Lin- murine bone marrow cells. All novel target genes examined displayed differential expression in shRNA transduced cells relative to an empty vector control (Fig 7), which is consistent with the observed array data as well as the hypothesis that AhR is a direct regulator of these genes in hematopoietic cells. The viral construct used was observed to yield significant reduction of AhR mRNA in Lin- cells and significant reduction of AhR protein levels in Hepa1c1c7 cells. In addition, 80% of cells were successfully transduced as indicated by the expression of GFP (S5 Fig)

## Discussion and Conclusions

The data presented here suggest a role for the AhR in regulating gene expression in LT-HSCs during aging. Importantly, we identified several novel target genes that AhR may directly regulate during aging of the hematopoietic system. We previously reported that lack of AhR produces a myeloproliferative phenotype in aging mice [6]. The present study identifies some underlying gene expression changes that may contribute to this phenotype. Consistent with our hypothesis that AhR is an important regulator of HSC gene expression, GSEA revealed a significant enrichment in LT-HSCs from AhR-KO mice. Many of these enriched genes are known/suspected to play a role in the regulation of HSC function (S3 Table). These include HoxB13 (a proposed tumor suppressor with described roles in cancer variety of tumor types) [28–30], CDKN1A (p21, a cyclin dependent kinase inhibitor that has been shown to be important in the long term maintenance of HSCs during stress, such as aging or serial transplant) [31], PLK4 (recently found to be deregulated in 70% of human lymphoma or myelodysplasia /leukemia clinical samples) [32] and Runx1t1 (known to play a role in human acute myeloid leukemia) [33, 34].

These data are consistent with the proliferative phenotype observed in AhR-KO mice, and further support the hypothesis that AhR regulates the expression of genes involved in HSC proliferation and function. If lack of AhR promotes a proliferative phenotype in HSCs, this could lead to enhanced loss of hematopoietic capacity and/or lineage skewing at earlier ages than would be expected. Abnormal proliferation of HSCs can lead to premature exhaustion of the hematopoietic system [35–39], or an abnormal skewing towards particular lineages of blood cells with increasing age [40, 41]. These conditions are potentially permissive for the development of hematological diseases such as myelodysplasia, aplastic anemia and leukemia. Genes regulated by AhR during chronological aging of murine HSCs provide insight into the pathways that are altered due to chronological and replicative aging of hematopoietic stem cells in AhR-KO mice, and the mechanisms underlying subsequent development of myeloproliferation or premature HSC exhaustion. Our lab has previously reported these phenotypes in AhR-KO mice [5, 6].

In order to identify altered genes that are affected by both lack of AhR expression AND age, we utilized a reductive strategy to exclude genes that are altered with age alone in either AhR-KO mice or WT mice. Pathway analysis of the remaining genes (genes that require age and lack of AhR) indicated that they may be involved in cell functions such as Cancer, Cell Growth and Proliferation, and Hematological System Development and Function. A subset of the latter genes was selected based on the presence of a putative AhRE for validation by qPCR. The genes differentially expressed included PDGF-D, Smo, Wdfy1, Zbtb37 and Zfp382. While the observed relative expression changes in these genes in vivo and in vitro following knockdown of AhR were not exactly the same, the direction of the changes were consistent. This is consistent with the hypothesis that AhR does regulate these genes, but also suggests that additional mechanisms may influence the expression values observed in vivo. Wdfy1, Zbtb37 and Zfp382 are largely undescribed in hematopoiesis and as such represent novel candidate target genes for AhR regulation of HSC function. Additional work is needed to define the exact role of these genes in HSC biology and their relationship to AhR.

PDGF-D is a member of the platelet-derived growth factor family, and has described roles in certain cancers but is largely under-studied as a regulator of hematopoiesis. Smo (Smoothened) is a member of the sonic hedgehog signaling (Shh) pathway, and both stromal and HSC intrinsic Shh signaling has been implicated as an important regulator of hematopoiesis [42–44]. It has been reported that human CD34+ cord blood HSCs express Smo and that treatment of HSCs with Shh ligand stimulates the proliferation of HSCs while preserving their engraftment potential into immune-compromised mice [45]. However, the role of Smo in regulating definitive adult hematopoiesis is controversial, as an inducible Smo-KO mouse doesn’t appear to display a hematopoietic phenotype [46–48]. This does not necessarily preclude the possibility that Smo is important for development of the hematopoietic system, but does suggest that it is dispensable for adult hematopoiesis. It is possible that Smo, or downstream effectors Gli1-3, do have regulatory effects on hematopoiesis, but only in the context of aging and through interaction with other genes that we observed to have differential expression in aged AhR-KO mice.

In order to determine functional overlap with AhR, Pathway Analysis was performed using Ingenuity. Our IPA network builds which included our 5 novel AhR targets (PDGF-D, Smo, WDFY1, Zbtb37 and Zfp382) as well as described AhR functions, revealed overlap between biological functions such as number of hematopoietic progenitor cells, number of leukocytes, and self-renewal of hematopoietic progenitors (Fig 5, S2, S3 and S4 Figs). These proposed biological functions are consistent with the hypothesis that AhR regulates the output and function of hematopoietic cells, in part, by regulating the expression of PDGF-D, Smo, Wdfy1, Zbtb37 and Zfp382. Lentiviral-mediated knockdown of AhR in primary murine Lin- cells induced changes in expression of all 5 candidate AhR targets, which is consistent with the hypothesis that AhR directly regulates these genes in hematopoietic cells. The fact that many genes were identified whose differential expression is dependent on both aging and lack of AhR expression (based on comparisons with our previously published array data in young animals) is consistent with the hypothesis that a lifetime lack of AhR ultimately dysregulates HSC signaling. Furthermore, the deficiency in AhR expression contributes to the development of hematopoietic phenotypes such as myeloproliferation, premature exhaustion and impaired engraftment in transplantation assays. It is important to note that lack of stromal AhR signaling in non-hematopoietic cells (within bone marrow or distant tissues of AhR-KO mice) could act in a paracrine or endocrine manner to alter gene expression in hematopoietic cells. The role of AhR in the hematopoietic niche is largely unstudied, but a recent publication did find that stromal cells with low AhR expression are not supportive for HSC function [49]. It is also possible that there are sex-dependent differences in HSC gene expression, and the present study only examined female mice. Additional work is needed to confirm that these novel genes are directly regulated by AhR in an HSC intrinsic fashion, and the mechanisms by which these alterations produce changes in HSC function or output. In addition, studies designed to test the role of the novel AhR regulatory target genes suggested by this work should be performed, in order to elucidate the mechanisms by which changes in PDGF-D, Smo, Wdfy1, Zbtb37 and Zfp382 expression can alter hematopoietic stem cell function and contribute to the phenotypes reported in AhR-KO mice.

## Supporting Information

S1 ARRIVE Checklist ARRIVE Checklist. This work has been described in accordance with the ARRIVE guidelines (Animal Research: Reporting of In Vivo Experiments).
(PDF)

S1 Fig Gating strategy for isolation of LSK-SLAM+ cells. Lin- cells from WT or AhR-KO mice were isolated and stained for CD34 (Red B), cKit (Green A), Sca1 (Violet H), CD48 (Blue B) and CD150 (Red C). Events were acquired on a BD LSR11 18-color Flow Cytometer. Events were gated using geometric gating to exclude doublets. A gate was applied to CD34- events. CD34-LSK+ cells were then gated on CD48-CD150+ to identify LT-HSCs (LSK-SLAM+).
(TIF)

S2 Fig Functional overlap of AhR and qPCR validated novel target genes in cancer. The subset of genes whose differential expression was dependent on age and lack of AhR expression, and subsequently validated by qPCR (PDGF-D, Smo, Wdfy1, Zbtb37 and Zfp382), was analyzed to determine possible functional overlap with the AhR in the context of biological functions and properties of cancer.
(TIF)

S3 Fig Functional overlap of AhR and novel target genes in cell proliferation. The subset of genes whose differential expression was dependent on age and lack of AhR expression, and subsequently validated by qPCR (PDGF-D, Smo, Wdfy1, Zbtb37 and Zfp382), was analyzed to determine possible functional overlap with the AhR in the context of cell proliferation, which is an important aspect of the regulation of hematopoietic stem cell function and maintenance.
(TIF)

S4 Fig Functional overlap of AhR and novel target genes in cell migration. The subset of genes whose differential expression was dependent on age and lack of AhR expression, and subsequently validated by qPCR (PDGF-D, Smo, Wdfy1, Zbtb37 and Zfp382), was analyzed to determine possible functional overlap with the AhR in the context of cell migration.
(TIF)

S5 Fig Extent of reduction in AhR mRNA and protein following lentiviral transduction. Lin- bone marrow cells were transduced as described, and the percent of GFP positive cells was determined. (A) Representative fluorescent profiles for a single untransduced and transduced sample. (B) Summary data for transduction efficiency (n = 3). (C) Level of reduction of AhR mRNA in lin- cells (n = 2) (D) extent of reduction of AhR protein in hepatoma cells using various shRNA clones. TRC0000218025 was determined to yield >90% reduction. AhR levels were normalized to the beta-actin band for each sample loaded.
(TIF)

S1 Table Top 25 up-regulated genes found to be differentially expressed in aged AhR-KO mice. (PDF)

S2 Table Top 25 down-regulated genes found to be differentially expressed in aged AhR-KO mice. (PDF)

S3 Table Top 20 enriched HSC-aging related genes. The gene set analyzed was previously found to be altered in aging HSCs and reported to the Broad Institute Molecular Signatures Database. AhR-KO mice display alterations in this gene set relative to WT controls.
(TIF)
