# Supplementary figures and images for: Deficiency in Aryl Hydrocarbon Receptor (AHR) Expression throughout Aging Alters Gene Expression Profiles in Murine Long-Term Hematopoietic Stem Cells
Source: PLoS One. 2015 Jul 24;10(7):e0133791. doi: 10.1371/journal.pone.0133791 (PMC4514744; doi:10.1371/journal.pone.0133791)

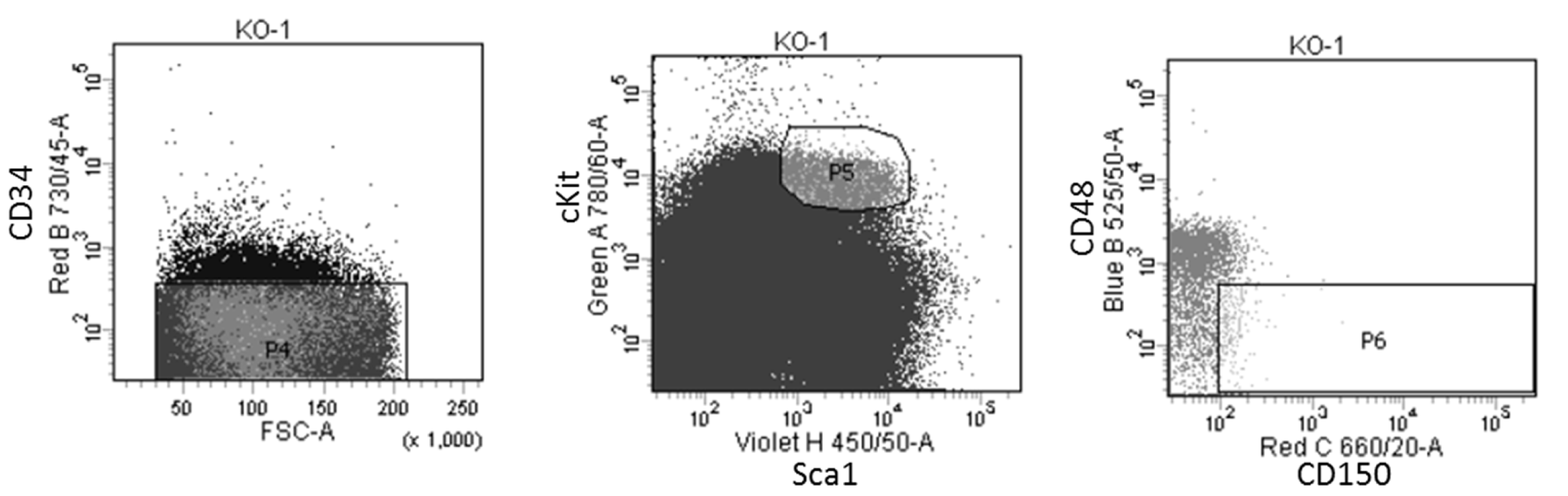

Supplement: S1 Fig — Lin- cells from WT or AhR-KO mice were isolated and stained for CD34 (Red B), cKit (Green A), Sca1 (Violet H), CD48 (Blue B) and CD150 (Red C). Events were acquired on a BD LSR11 18-color Flow Cytometer. Events were gated using geometric gating to exclude doublets. A gate was applied to CD34- events. CD34-LSK+ cells were then gated on CD48-CD150+ to identify LT-HSCs (LSK-SLAM+). (TIF) [file pone.0133791.s002.tif]

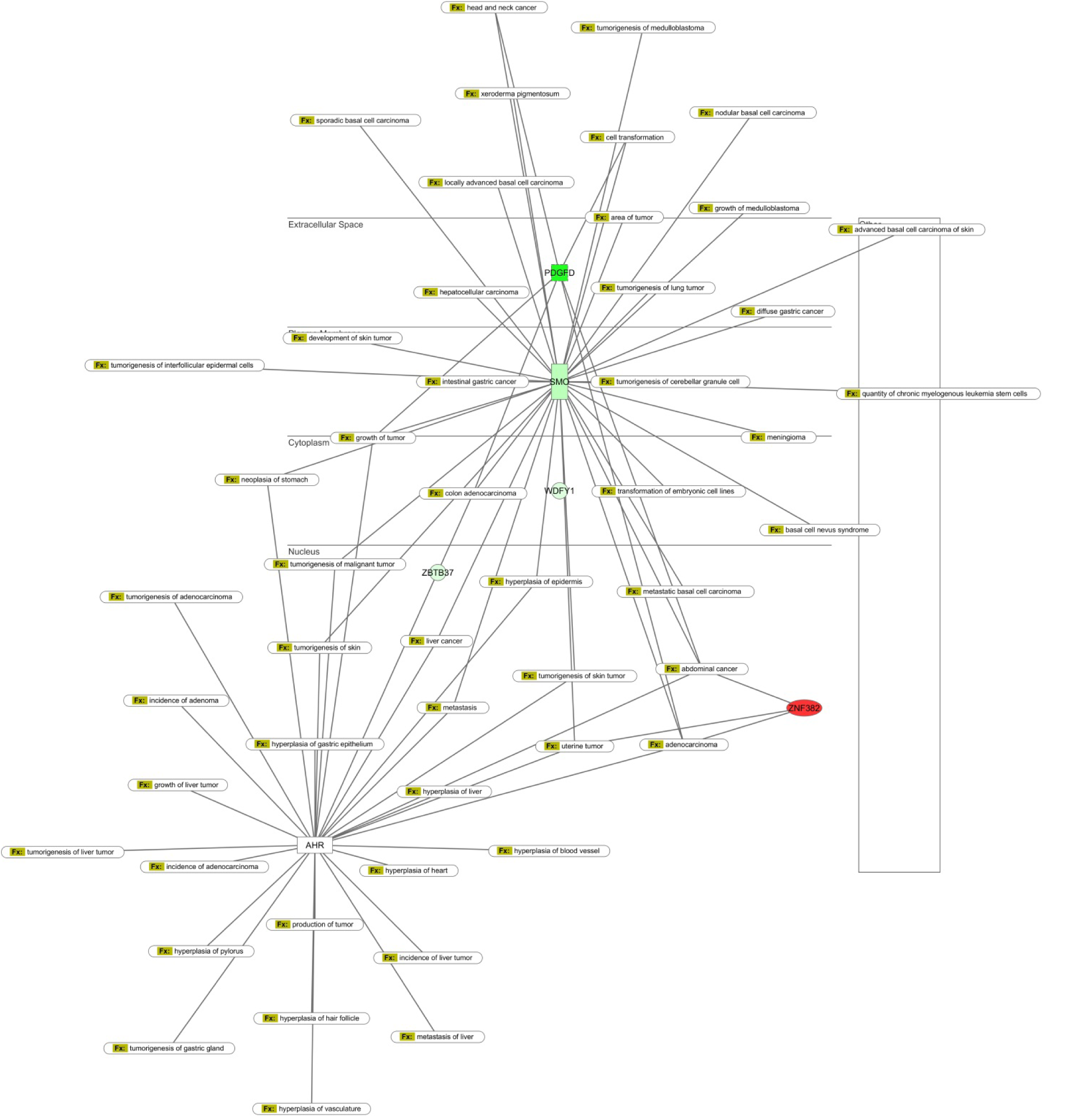

Supplement: S2 Fig — The subset of genes whose differential expression was dependent on age and lack of AhR expression, and subsequently validated by qPCR (PDGF-D, Smo, Wdfy1, Zbtb37 and Zfp382), was analyzed to determine possible functional overlap with the AhR in the context of biological functions and properties of cancer. (TIF) [file pone.0133791.s003.tif]

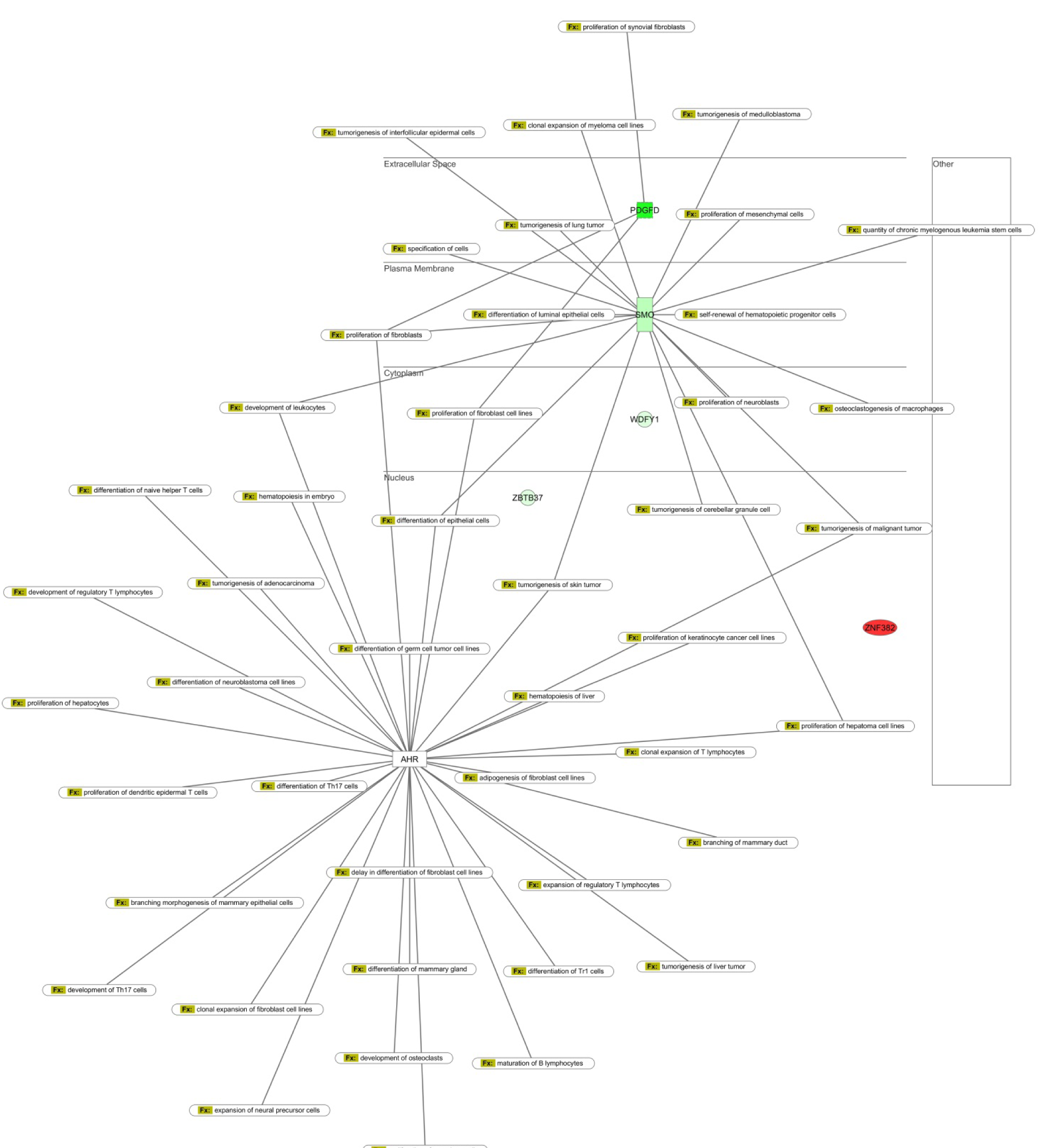

Supplement: S3 Fig — The subset of genes whose differential expression was dependent on age and lack of AhR expression, and subsequently validated by qPCR (PDGF-D, Smo, Wdfy1, Zbtb37 and Zfp382), was analyzed to determine possible functional overlap with the AhR in the context of cell proliferation, which is an important aspect of the regulation of hematopoietic stem cell function and maintenance. (TIF) [file pone.0133791.s004.tif]

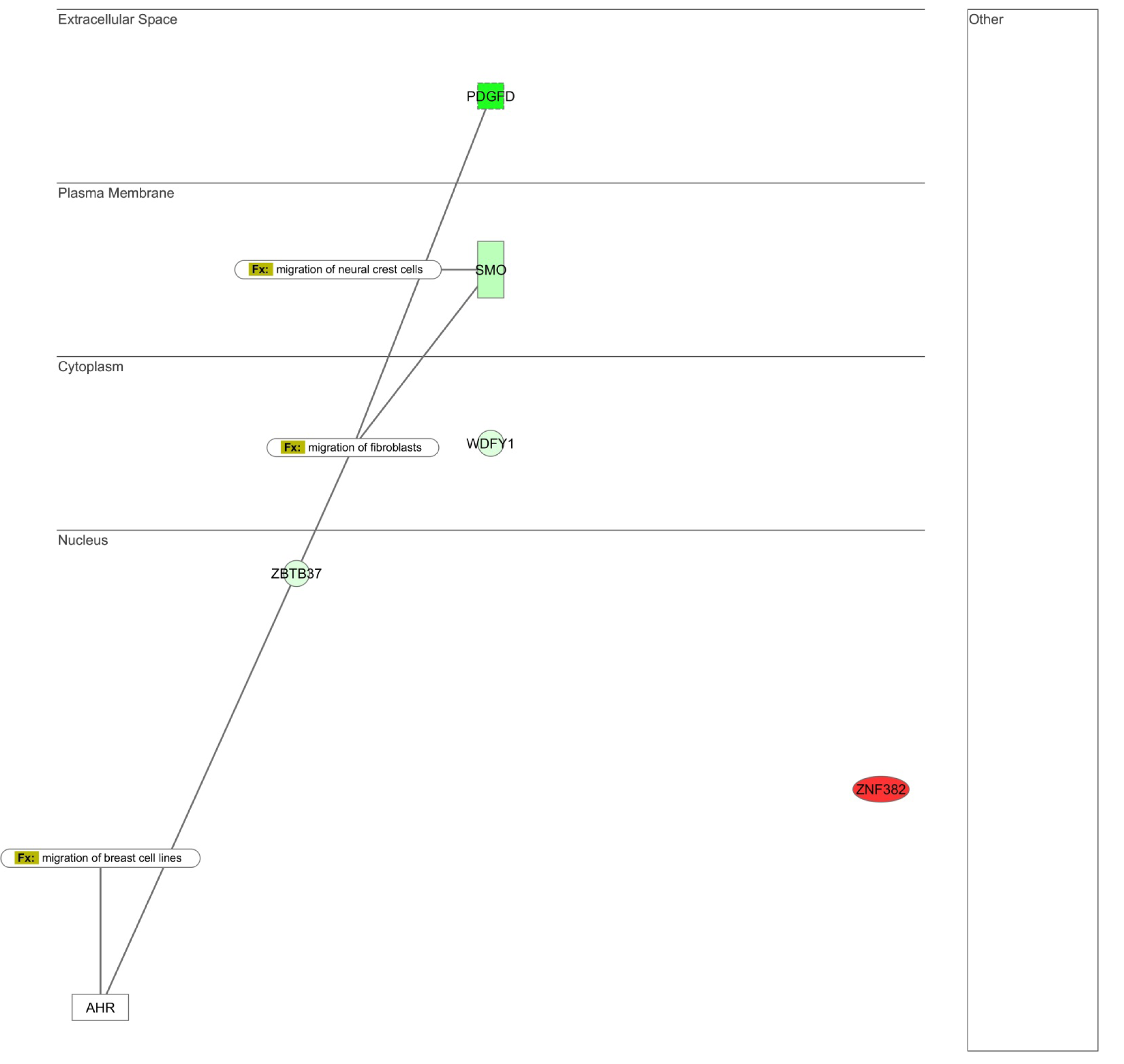

Supplement: S4 Fig — The subset of genes whose differential expression was dependent on age and lack of AhR expression, and subsequently validated by qPCR (PDGF-D, Smo, Wdfy1, Zbtb37 and Zfp382), was analyzed to determine possible functional overlap with the AhR in the context of cell migration. (TIF) [file pone.0133791.s005.tif]

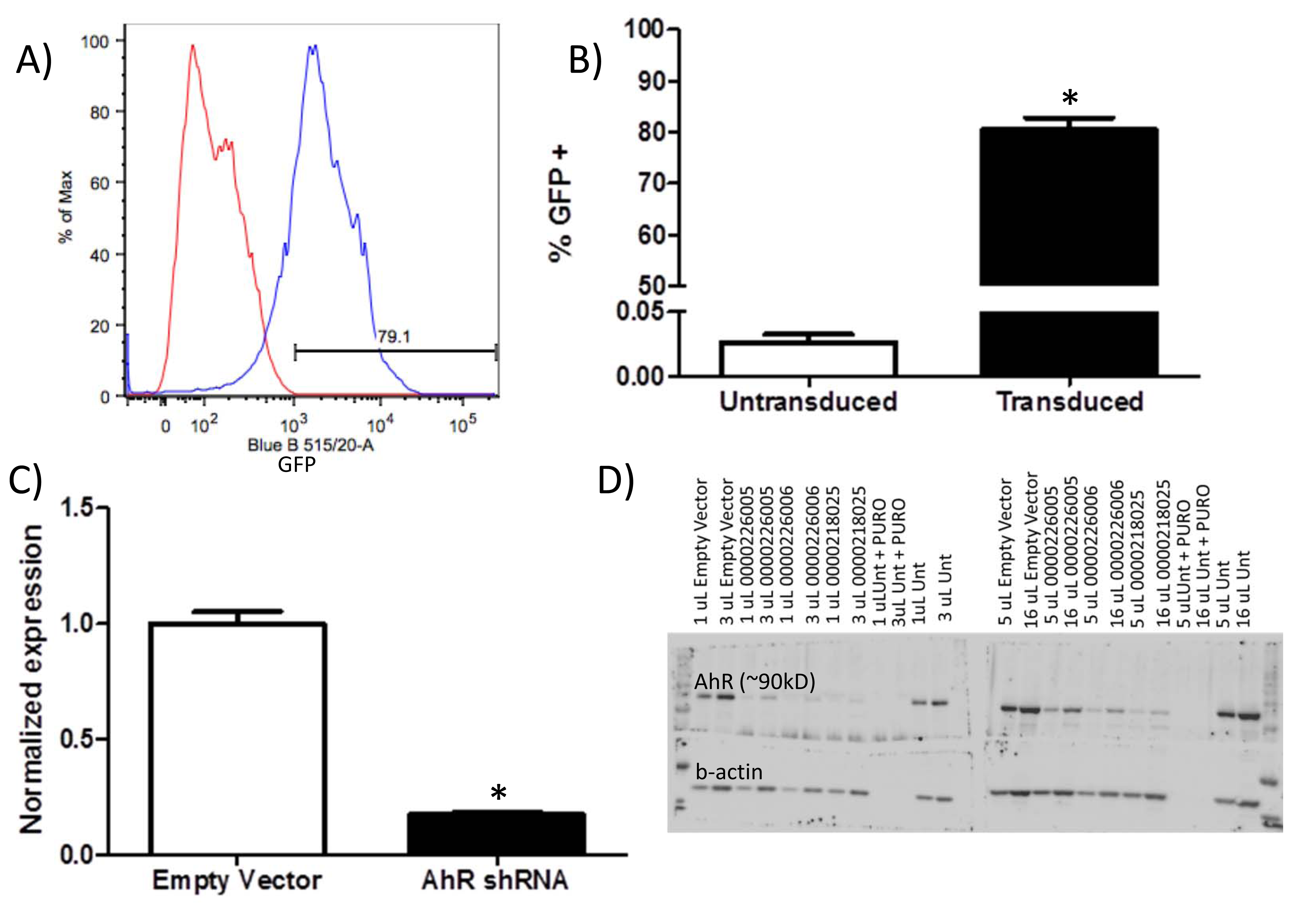

Supplement: S5 Fig — Lin- bone marrow cells were transduced as described, and the percent of GFP positive cells was determined. (A) Representative fluorescent profiles for a single untransduced and transduced sample. (B) Summary data for transduction efficiency (n = 3). (C) Level of reduction of AhR mRNA in lin- cells (n = 2) (D) extent of reduction of AhR protein in hepatoma cells using various shRNA clones. TRC0000218025 was determined to yield >90% reduction. AhR levels were normalized to the beta-actin band for each sample loaded. (TIF) [file pone.0133791.s006.tif]

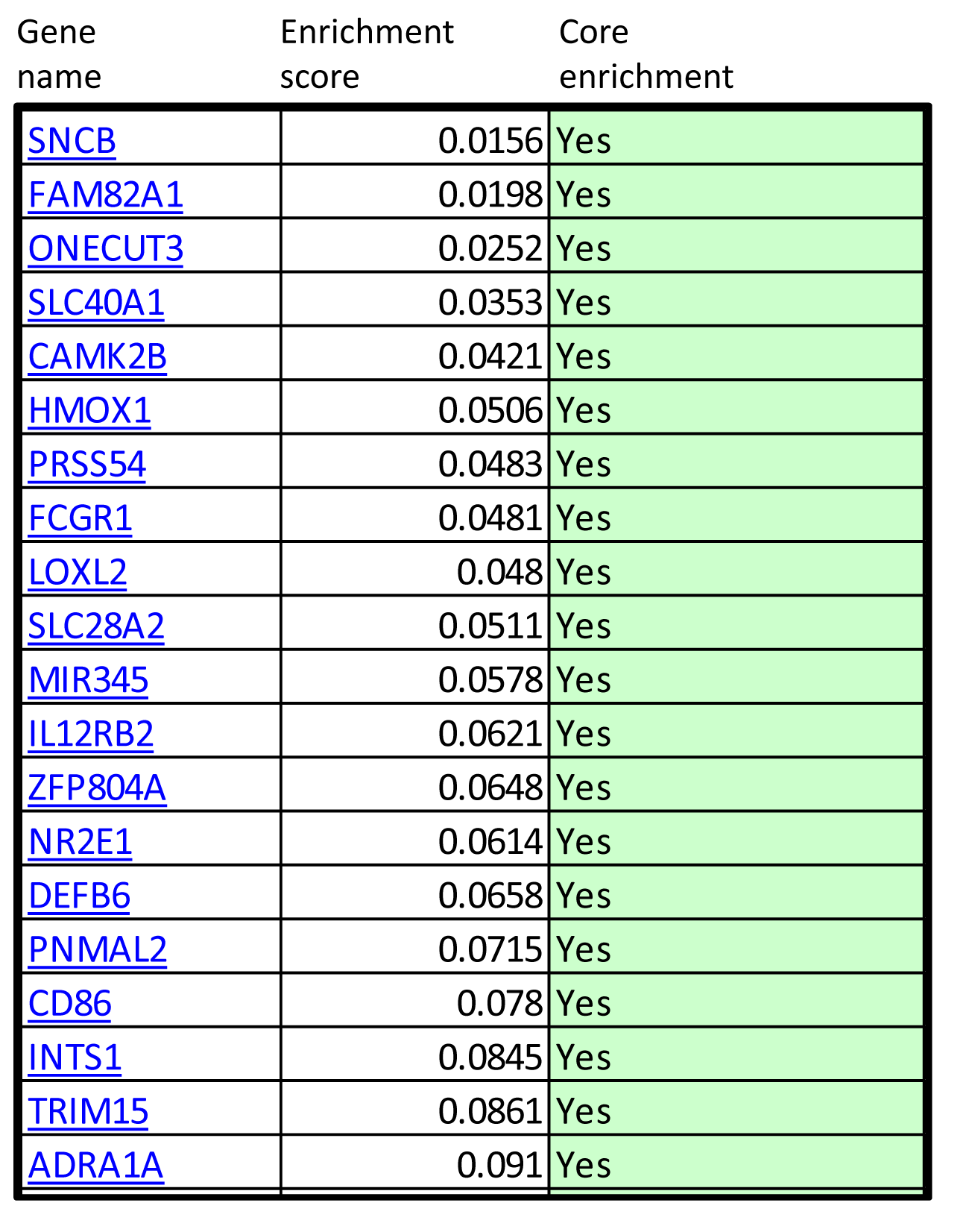

Supplement: S3 Table — The gene set analyzed was previously found to be altered in aging HSCs and reported to the Broad Institute Molecular Signatures Database. AhR-KO mice display alterations in this gene set relative to WT controls. (TIF) [file pone.0133791.s009.tif]
