# Supplementary material for: Deficiency in Aryl Hydrocarbon Receptor (AHR) Expression throughout Aging Alters Gene Expression Profiles in Murine Long-Term Hematopoietic Stem Cells
Source: PLoS One. 2015 Jul 24;10(7):e0133791. doi: 10.1371/journal.pone.0133791 (PMC4514744; doi:10.1371/journal.pone.0133791)
Supplement: S1 Table — (PDF) [file pone.0133791.s007.pdf]

Table 1. The top 25 differential up-regulated genes expression in Aging AhR-KO mice.

Gene	Gene title	Location	Transcript ID	Fold change	P Values<
Gm1553	predicted gene 1553	chr10C1	NC_000076.6	9.003	0.027
Gm10319	predicted gene 10319	chr6	NR_003624.2	6.691	0.049
Olfr832	olfactory receptor 832	chr9	NM_001011824	5.968	0.032
Mup10	major urinary protein 10	chr4	OTTMUST00000017162	5.673	0.036
Mir130a	microRNA 130a	chr2	NR_029544	4.334	0.041
Olfr891	olfactory receptor 891	chr9	NM_146478	4.110	0.042
Mgll	monoglyceride lipase	chr6	OTTMUST00000058400	4.028	0.043
Sult2a3	sulfotransferase family 2A, dehydroepiandosterone - preferring, member 3	chr7	OTTMUST00000051842	3.926	0.035
AF357341	snoRNA AF 357341	chr12	ENSMUST00000083088	3.523	0.004
Mir20b	microRNA	chromosome x	NR_030273	3.359	0.024
Gm11562	predicted gene 11562	chr11	OTTMUST00000004420	3.356	0.037
Wbp2nl	WBP2 N-terminal like	chr15	NM_029066	3.066	0.033
Hoxb13	homeobox B13	chr11	OTTMUST00000003919	3.047	0.001
Slco4c1	solute carrier organic transporter Family, member 4C1	chr1	NM-172658	2.920	0.028

Gm5431	predicted gene 5431	chr11	OTTMUST00000012286	2.897	0.022
Lama5	laminin, alpha 5	chr2	OTTMUST00000039036	2.664	0.017
Olfr945	olfactory receptor 945	chr9	NM_146506	2.651	0.031
Tas2r123	taste receptor, type 2, member 123	chr6	NM_207025	2.648	0.048
Lrrc55	leucine rich repeat containing 55	chr2	NM_001033346	2.643	0.020
Mir3068	microRNA 3068	chr12	NR_037228	2.631	0.043
Ces1f	carboxylesterase 1F	chr8	OTTMUST00000060178	2.553	0.041
Clec2g	C-type lectin domain family 2, member g	chr6	OTTMUST00000072343	2.504	0.014
Pik3r3	phosphatidylinositol 3 kinase, regulatory subunit, polypeptide 3 (p55)	chr3	OTTMUST00000021439	2.482	0.044
Olfr450	olfactory receptor 450	chr6	NM_146445	2.390	0.007
Atp6v0d2	ATPase, H+ transporting, lysosomal VO subunit D2	chr4	OTTMUST00000014512	2.379	0.034
