# Supplementary material for: Deficiency in Aryl Hydrocarbon Receptor (AHR) Expression throughout Aging Alters Gene Expression Profiles in Murine Long-Term Hematopoietic Stem Cells
Source: PLoS One. 2015 Jul 24;10(7):e0133791. doi: 10.1371/journal.pone.0133791 (PMC4514744; doi:10.1371/journal.pone.0133791)
Supplement: S2 Table — (PDF) [file pone.0133791.s008.pdf]

Table 2. The top 25 differential down-regulated genes expression in Aging AhR-KO mice.

Gene	Gene title	Location	Transcript ID	Fold change	P Value
Olfr293	olfactory receptor 293	chr7	OTTMUST00000097514	61.139	0.019
Lce3f	late cornified envelope 3F	chr3	NM_001018079	40.714	0.027
Mir202	microRNA 202	chr7	NR_029589	9.629	0.037
Mir1946b	microRNA	chr9	NR_035496	6.835	0.033
Prl2c5	prolactin family 2, subfamily C, member 5	chr13	OTTMUST00000068578	5.771	0.045
Olfr1154	olfactory receptor 1154	chr2	OTTMUST00000033077	5.591	0.019
Olfr867	olfactory receptor 867	chr9	NM_001011748	5.465	0.036
Vmn1r227	vomeronasal 1 receptor 227	chr17	OTTMUST00000103503	4.564	0.025
Adam34	a disintegrin and metallopeptidase Domain 34	chr8	NM_145745	4.417	0.043
Mir300	microRNA 300	chr12	NR_029651	4.186	0.001
Hist3h2ba	histone cluster 3, H2ba	chr11	OTTMUST00000012785	4.175	0.031
Olfr1283	olfactory receptor 1283	chr2	OTTMUST00000035778	3.523	0.015
Olfr918	olfactory receptor 918	chr9	NM_146375	3.480	0.016
Bpifc	BPI fold containing family C	chr10	OTTMUST00000051766	3.413	0.014

Olfr781	olfactory receptor 781	chr10	NM_146728	3.276	0.042
Gm10471	predicted gene 10471	chr5	OTTMUST00000093863	3.196	0.015
Nlrp4d	NLR family, pyrin domain containing 4D	chr7	OTTMUST00000113575	3.095	0.043
Gm19984	predicted gene 19984	chr11	NC_000077	3.056	0.028
Fhl4	four and a half LIM domain	chr10	NM_010214	3.039	0.028
Xlr3b	x-linked lymphocyte-regulated 3B	chromosome x	OTTMUST00000042855	2.970	0.017
Olfr801	olfactory receptor 801	chr10	NM_146285	2.889	0.026
Vmn1r208	vomeronasal 1 receptor 1156	chr13	OTTMUST00000001102	2.709	0.014
Olfr1156	olfactory receptor 156	chr2	OTTMUST00000033082	2.700	0.028
Spt1	salivary protein 1	chr15	ENSMUST00000037685	2.64	0.018
Olfr1045	olfactory receptor 1045	chr2	OTTOMUST00000032800	2.482	0.008
